# Toxicity and Toxicokinetics of a Four-Week Repeated Gavage of Levamisole in Male Beagle Dogs: A Good Laboratory Practice Study

**DOI:** 10.3390/ph17010141

**Published:** 2024-01-22

**Authors:** Jiahui Zhang, Junxiang Wang, Lingfan Chen, Xiangbin Yu, Shuihua Zhang, Yue Yu

**Affiliations:** 1 School of Pharmacy, Fujian Medical University, Fuzhou 350122, China; barolxicarrel@163.com (J.Z.); 18846834706@163.com (J.W.); lingfanchen@163.com (L.C.); yxb4666@fjmu.edu.cn (X.Y.); 2 Fujian Center for New Drug Safety Evaluation, Fuzhou 350122, China

**Keywords:** levamisole, repeated dose toxicity, toxicokinetics, NOAEL, beagle dogs

## Abstract

Levamisole (LVM) is considered an immunomodulatory agent that has the potential to treat various cancer and inflammation diseases. However, there is still much debate surrounding the toxicokinetic and toxicological information of LVM. Therefore, it is crucial to assess its toxicity to provide useful data for future human LVM risk assessments. In this study, a barrier environment was established under the guidance of good laboratory practice (GLP) at the Fujian Center for New Drug Safety Evaluation. Male beagle dogs were orally administered with 5, 15, and 30 mg/kg of LVM daily for four weeks. Toxicity assessment was based on various factors such as mortality, clinical signs, food and water consumption, body weight, body temperature, electrocardiogram, ophthalmological examination, hematology, serum biochemistry, organ/body coefficients, histopathological study, and toxicokinetic analysis. The results of this study showed that LVM did not exhibit any significant toxicological effects on beagle dogs at the exposure levels tested. A no observed adverse effect level (NOAEL) of LVM was set at 30 mg/kg/day for male beagle dogs, which is equivalent to a 12-fold clinical dose in humans. Moreover, the repeated exposure to LVM for four weeks did not lead to any bioaccumulation. These findings provide valuable insights for future human LVM risk assessments.

## 1. Introduction

Levamisole (LVM) has been widely recognized as an immunomodulatory agent that could potentially be utilized to treat various cancer and inflammation-related diseases [1,2]. LVM is capable of inducing the secretion of various cytokines, which in turn, promotes the differentiation and proliferation of T-cells, increases the levels of cyclic guanylate, strengthens the phagocytosis function of mononuclear macrophages, improves the overall immunity of the body, and restores abnormal immune function to normal levels.

Currently, LVM has been widely applied as an anti-helminthic agent in veterinary medicine [3]; however, studies on humans have reported potential risks associated with LVM exposure, which induced the withdrawal of LVM from the United States market in 2000. Reports involved complications associated with leukoencephalopathy, neutropenia, agranulocytosis, skin necrosis, etc. [4], among which, leukoencephalopathy has been well documented as a life-threatening complication. Despite often being reversible with cessation of LVM exposure [5], the potential risk is still a concern when using LVM clinically. Over the last decade, LVM has been increasingly encountered as a cutting agent in cocaine, because of its look in a white powder with a “fish scale”, similar to cocaine. As reported [6], LVM could be metabolized to a substance with amphetamine-like psychostimulatory properties and a long half-life in vivo, through which, LVM potentiates and prolongs the stimulatory effects of cocaine. Some reports indicated that the toxicology of LVM was mainly from being co-administrated with cocaine [7]. There is still an estimated 65% of cocaine samples worldwide that have the presence of LVM [8].

After reviewing the complications associated with LVM, it is interesting to note that almost all reported side effects were associated with oral administration of the drug [9,10,11]. Furthermore, the safe dosage of LVM for both human and animal use remains controversial. This could potentially be due to safety evaluations that were not conducted in compliance with good laboratory practice (GLP). Therefore, it is imperative to determine the precise no observable adverse effect level (NOAEL) of LVM before it can be widely reintroduced into clinical settings for patient use.

Toxicokinetics is the scientific study of the kinetics involved in the absorption, distribution, metabolism, and excretion of a xenobiotic in the context of toxicity evaluation. The primary objective of conducting toxicokinetic studies is to determine the precise rate, extent, and duration of systemic exposure of a test animal species to a test compound at different dose levels employed during toxicity studies. This data is crucial as it provides a basis for direct comparison with human exposure to the test compound [12,13].

**Figure 1:**
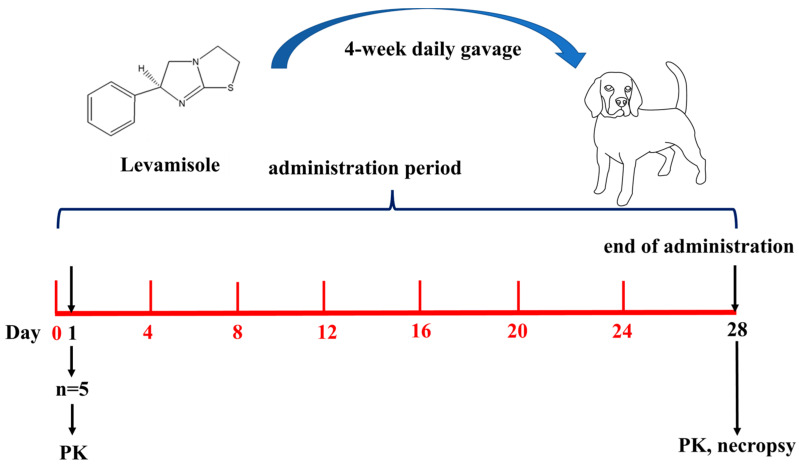
The experimental scheme.

We strictly follow GLP, the quality assurance system, to ensure the integrity, consistency, and reproducibility of non-clinical studies and tests. To evaluate the systemic toxicity of LVM and investigate its NOAEL value, here in this study, we established a barrier environment under GLP, and beagle dogs were gavaged with LVM daily for 4 weeks. The toxic reactions and the reversibility of its potential damage were recorded to study the toxicity. Meanwhile, the C_max_ and AUC values were calculated to study the toxicokinetics of LVM in beagle dogs. The whole experimental scheme is shown below (Figure 1):

## 2. Results

### 2.1. Clinical Observation

**Figure 2:**
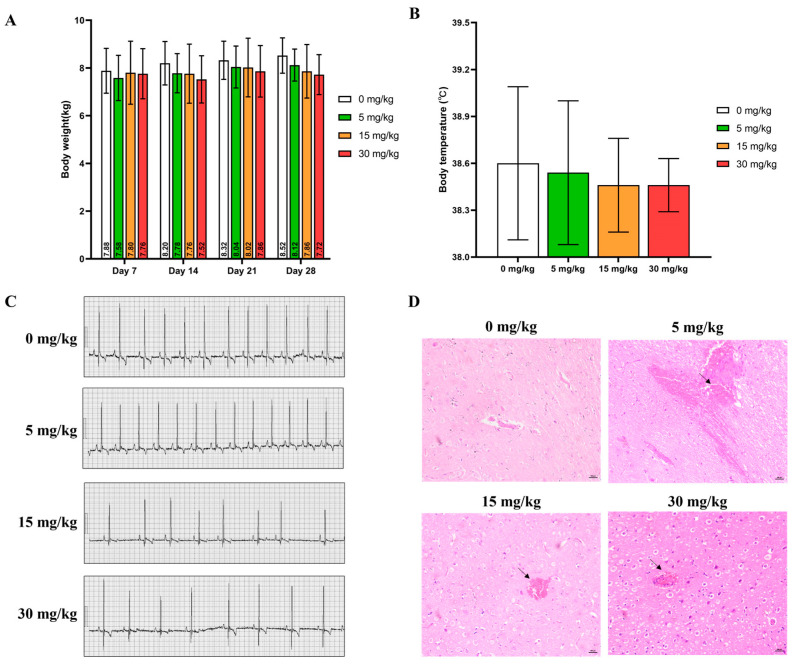
(**A**) Body weight, (**B**) Body temperature, and (**C**) Representative ECG patterns of beagle dogs during the experimental course (mean ± SD, n = 5). (**D**) Typical images of H&E staining of the brain acquired at 200× magnification (Scale bar = 100 μm). Arrow: bleeding.

All experimental animals survived the 28-day experimental period. No signs of severe abnormality were found during the whole course of administration except for animals in the 30 mg/kg group, which showed vomiting, tremor, convulsions, hypoactivity, and gait disorder soon after gavage (Clinical observations for individual animals were shown in Table S1). Compared with the control group over the same period, no significant statistical difference in the average body weight (Figure 2A), body temperature (Figure 2B), and electrocardiogram indexes (Figure 2C) of all experimental animals were calculated (*p* > 0.05). Due to the toxic reactions such as tremors, convulsions, and gait disorders observed at high doses, pathological examination was performed on the brain. Minor brain hemorrhage could be observed in the 5, 15, and 30 mg/kg groups (Figure 2D) (The ECG values of individual experimental animals were shown in Table S2).

### 2.2. Hematological Examination

As indicated in Table 1, during the administration period, the counts for WBC and NEUT decreased significantly in all experimental animals that orally consumed 5 mg/kg LVM and 30 mg/kg LVM, while there is no significant difference in WBCs and NEUT in dogs given 15 mg/kg LVM relative to the control group. The MONO (%) showed a significant increase at the 30 mg/kg LVM dosage (*p* < 0.05). Additionally, LYMPH and EO exhibited significant decreases only at the 30 mg/kg LVM dosage compared to the control group (*p* < 0.05).

### 2.3. Blood Biochemical Indicators

The changes in TG, CHOL, and Na^+^ levels in male beagle dogs post LVM gavage were examined. The levels of TG showed significant changes followed by different doses of LVM compared to the control group. The Na^+^ level was significantly higher only in male beagle dogs administered with 5 mg/kg LVM while we found a prominent increase in CHOL level after a 15 mg/kg and 30 mg/kg dosage of LVM compared to the control group. Results are shown in Table 2.

### 2.4. Bone Marrow Examination

According to the results of bone marrow cytology, 1 animal in the control group had reduced bone marrow proliferation and 1 animal had abnormally active bone marrow proliferation. In the low-dose and medium-dose groups, no abnormal bone marrow proliferation was observed, and 2 animals in the high-dose group had reduced bone marrow proliferation. Individual data from bone marrow smears are shown in Table S3. There were no significant differences in the parameters of bone marrow smears among all the treatment groups when compared with the control group in Table 3.

### 2.5. Gross Anatomy

There was no general abnormality observed in each group of animals. Ophthalmological examination showed no abnormalities in the visual response, eyelid, conjunctiva, cornea, sclera, iris, pupil, lens, vitreous body, and fundus of all experimental animals during the whole experimental period. Then, organ/body weight (O/B) coefficients at day 28 were calculated, while the O/B coefficient of the thymus in all experimental groups showed a dose-response decrease compared with the control group (*p* < 0.05), and the O/B coefficient of epididymal was calculated higher (*p* < 0.05) than in the control group (Table 4).

### 2.6. Toxicokinetics Study of LVM

#### 2.6.1. Method Validation

**Figure 3:**
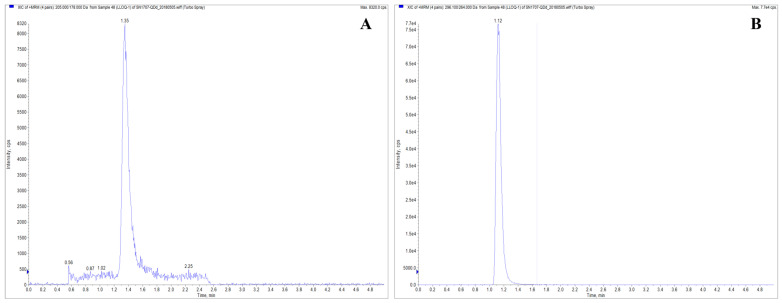
Chromatogram of LVM in plasma. The MRM transitions were 205.1→178.2 was monitored for the LVM ion pair (**A**), and 296.1→264.1 for the mebendazole ion pair (**B**).

This study established an LC-MS/MS method for determining levamisole hydrochloride in Beagle dog plasma and comprehensively validated the method. The results demonstrated that neither the analyte nor the internal standard exhibited any interference or residue in the plasma samples. The chromatogram of LVM in plasma is shown in Figure 3. The correlation coefficient of the standard curve was R ≥ 0.99, and the linear range was 0.5~200 ng/mL. The accuracy and precision results (Table 5) showed that the average accuracy of LLOQ was 91.1~108.3%, the intra-batch precision (RSD) was 2.61~13.45%, and the inter-batch precision was 12.00%. The average accuracy of low, medium, and high QC samples was 97.2~107.4%, the intra-batch precision (RSD) was 1.94~6.02%, and the inter-batch precision (RSD) was 2.85~5.16%, which met the requirements. The extraction recovery results showed that the extraction recovery of levamisole hydrochloride at low, medium, and high concentrations was 75.65~77.92%, with RSD in the range of 0.44~1.48%; the extraction recovery of the internal standard was 74.38~82.32%, with RSD in the range of 1.79~3.49%, which were acceptable.

The accuracy, precision, matrix effect, extraction recovery, dilution reliability, and other parameters all met the requirements. The reserve solution was stable at 4 °C for 1 day, and the drug-containing plasma was stable at room temperature for 4 h, at −80 °C for 99 days, and after 1 or 3 freeze–thaw cycles. The plasma samples were stable within 24 h after being placed in the automatic sampler following sample preparation. The reproducibility of the established method was good, and the results were stable and reliable according to the reanalysis of plasma samples.

#### 2.6.2. Pharmacokinetic Parameters

**Figure 4:**
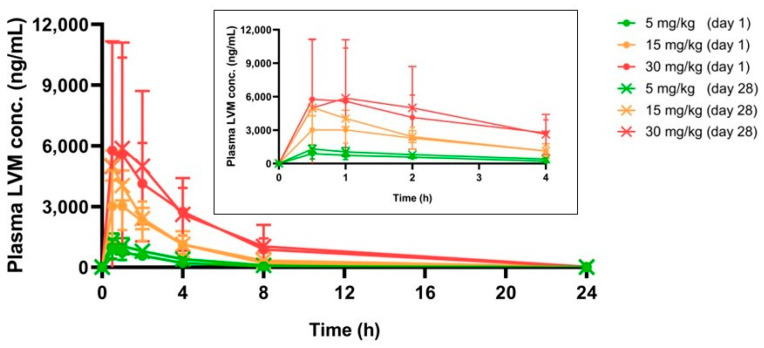
The real-time LVM plasma concentration in beagle dogs within 24 h, chart inserted was the real-time LVM plasma concentration in the first 4 h (mean ± SD, n = 5).

As shown in Table 6 and Figure 4, with the dose of LVM increased, C_max_ and AUC for LVM increased proportionally. The values of C_max_ (day 28)/C_max_ (day 1) and AUC (day 28)/AUC (day 1) in all experimental groups ranged from 0.77 to 1.41 and 1.08 to 1.62, respectively. In addition, 24 h post-administration, plasma LVM concentrations could not be observed at either day 1 or day 30. All results above indicated that oral administration of LVM at 5, 15, and 30 mg/kg showed no bioaccumulation during a 28-day regimen.

## 3. Discussion

GLP regulations are now an integral part of non-clinical drug development. In addition to ensuring the ethical treatment and welfare of animals, GLP can provide sufficient evidence of the validity, integrity, and reliability of non-clinical safety data. Most importantly, it provides an important safeguard for approval for use in human clinical studies. The study met the requirements of the GLP specification and agreed with the study director’s (SD) GLP declaration of conformity. We followed the SOPs of the experimental center and passed the quality assurance unit (QAU) review. All data documentation, including data collection, was carried out in full to comply with the SOP. Information and data were reviewed by SD at records. Any corrections and/or deviations were resolved prior to the release of the experimental report, ensuring the reliability and validity of the data.

The dosage design in this study was determined through the proposed clinical dose of LVM for humans (150 mg/day). Adult body weight is calculated as 60 kg, and the dose is equivalent to 2.5 mg/kg, which is equivalent to the dog dose of 4.6 mg/kg. In our previous study, results of a two-week preliminary test of oral gavage of LVM in beagle dogs showed that 1/2 of the animals in the 60 mg/kg dose group died on the 6th day after administration, and the rest developed tremors and convulsions on the 10th day after administration. Combined with the results of previous tests, the doses in this study were set at 5, 15, and 30 mg/kg on beagle dogs, which were equivalent to 2, 6, and 12-fold, respectively, of the proposed clinical dose.

The results of the toxicity study showed that LVM was well tolerated by experimental beagle dogs up to a dose of 30 mg/kg/day, as there was no significant systemic toxicity observed during the 4-week daily gavage. However, some symptoms such as vomiting, tremors, convulsions, hypoactivity, and gait disorders were noted, which may be related to the central nervous system and autonomic system, and further investigations are required. The study also indicated that LVM was well tolerated by experimental non-rodents with no significant toxicity observed.

The experimental dogs showed a dose-dependent restrained tendency in body weight gain, with the high-dose group showing the most significant effect. This suggests that oral administration of LVM could potentially decrease appetite in beagle dogs, leading to restrained body weight gain.

Certain diseases, including cancer, and certain treatments, including chemotherapy and radiation therapy, could cause immunosuppression, this is usually referred to as having a low white blood cell count [14,15]. However, these data fluctuated within the historical range and exhibited no dose or time-response relationships with physiological meaning. Small but significant changes in these parameters were observed. Significant changes in the number and percentage of WBC and NEUT observed at doses 5 mg/kg/day and 30 mg/kg/day in this study were likely related to indicate immunosuppression induced by LVM. However, these changes were not considered toxicologically significant in this study because values for WBC and NEUT still remained within the normal range of (6–17) × 10^9^ per liter for the WBC count and (3–12) × 10^9^ per liter for the NEUT count in a dog. Similar with the results of the hematological examination, the level change of MONO (%), LYMPH, EO, TG, and Na^+^ obtained in the blood biochemical examination could also be considered as no-toxicological significance as values were also within the limit of historical control data as reported [16,17]. Although a statistical difference in CHOL levels was observed between the 15 and 30 mg/kg groups compared to the negative control group, it appears that the increase approaching the clinical range was attributable to the rise in HDL, or ‘good’ cholesterol. This suggests that LVM may exert some influence on the CHOL values in beagle dogs without toxicological significance [18]. Also, blood indicators combined with organ coefficient indexes, we found that continuous administration of LVM did not affect liver and kidney indexes and weights, and it did not produce accumulation effects and toxicological effects in the kidneys and liver of beagle dogs [19].

Here in this study, the thymus showed a significant dose-dependent decrease, displaying harm from a high dose of LVM consumption. However, more attention should be paid to this fluctuation for safety reasons when orally administrated with LVM clinically. In addition, a swelling in the epididymis could also be observed in this study, which possibly indicated an epididymitis. As reported, this swelling could be intermittent and might go away by itself [20,21].

LVM showed rapid adsorption, distribution, metabolism, and excretion (ADME) processes after 5–30 mg/kg were orally administrated in a single dose, as the toxicokinetics study revealed a complete cleavage 24 h post gavage, regardless of the dose, number of repeat administrations, and administration duration. In addition, a notable dose-dependent exposure of LVM could be observed after gavage for AUC showed a significant increase in proportion to dose. However, no obvious changes in the AUC of LVM between day 1 and day 28, which indicated no bioaccumulation of LVM, could be observed after repeated administration in beagle dogs. The increase in dose did not result in stricter exposure [22]. Our research findings established an LC-MS/MS assay method for the determination of levamisole hydrochloride in beagle-dog plasma and may contribute to understanding the toxicokinetic properties following high exposure to LVM administration in order to support further clinical development of LVM.

## 4. Materials and Methods

### 4.1. Chemicals

Levamisole (S-6-Phenyl-2,3,5,6-tetrahydroimidazo [2,1-b] [17] thiazole, CAS no. 240-654-6), with a purity of 98% was purchased from Sigma-Aldrich (St. Louis, MO, USA). Mebendazole (5-Benzoyl-2-benzimidazolecarbamic acid methyl ester, CAS no. 31431-39-7), with a purity of 99.6%, applied as internal standard, was obtained from Shanghai Aladdin Biochemical Technology Co., Ltd. (Shanghai, China). Ammonium acetate and acetonitrile were both of analytical grade and purchased from Macklin Industrial Corporation (Shanghai, China).

### 4.2. GLP Barrier Environment

This experiment was conducted in accordance with the “Drug Non-clinical Research Quality Management Standards, Order No. 34” issued by the State Food and Drug Administration (SFDA) and “Technical Guidance on Toxicity Testing of Repeated Drug Administration Principles, 2014”. The studies mentioned in this article were approved by the Animal Ethics Committee of Fujian Medical University (APVL No: FJMU IACUC 2021-0039) and were conducted in accordance with the NIH Guide for the Care and Use of Laboratory Animals (8th edition). Beagle dogs (male, aged 6–7 months, weighing between 5.5–8.8 kg) were purchased from Nanjing Yadong Experimental Animal Research Center and were housed in stainless steel cages that complied with the minimum space requirements for experimental animals as outlined in the National Standard of the People’s Republic of China. The animals were kept in a facility equipped with a 12-h light-dark cycle, constant humidity (55 ± 10%), and temperature (24 ± 2 °C). Prior to the experiment, each animal was given a unique temporary number and was quarantined for 15 days. During this quarantine period, the animals were closely observed for their consumption and overall health.

The drinking water used in this study met the standards outlined in “Sanitation Standards for Drinking Water” in terms of microbial indicators, toxicological indicators, and general physical and chemical indicators. Moreover, the chow used in this study complied with “Nutrition Composition of Experimental Animal Compound Feed”.

### 4.3. Administration Regimen

The experimental beagle dogs were divided into four groups, each containing five animals, in accordance with Standard Operating Procedure 309A of Fujian Medical University. The doses administered were 5, 15, and 30 mg/kg.

The administration of LVM in this study followed the Standard Operating Procedure 108A (gavage administration) of Fujian Medical University. Gavage administration was chosen as it aligns with the proposed clinical route. In the study, LVM was dissolved in ultrapure water and administered as a solution at a rate of 5 mL/kg. The control group received an oral dose of ultrapure water. The dosing frequency was once per day for a duration of 4 weeks.

### 4.4. Subchronic Oral Toxicity Study

#### 4.4.1. Clinical Observations and Mortality

Observation of animals was conducted twice a day after gavage. The observation included, but was not limited to, survival, eyes, ears, nose, oral cavity, fur, abdomen, vulva, perianal, limbs, claws, flesh toes, gait, behavior, excretion, feeding, drinking, etc.

#### 4.4.2. Body Weight

The experimental beagle dogs were weighed once a week. On the day of necropsy, dogs were fasted for 12 h before the necropsy was performed to calculate organ and brain coefficients.

#### 4.4.3. Food and Water Consumption

All the animals herein have free access to food and water during the experimental period. Feed twice a day, morning and afternoon, giving 100–150 g per dog each time based on their weight. After gavage, the food intake of the animals was recorded once per day.

#### 4.4.4. Body Temperature

The body temperatures of all experimental animals were measured 28 days after the first administration.

#### 4.4.5. Electrocardiogram

The electrocardiogram was recorded 28 days after the first administration. Measurement indicators included heart rate (HR), PR interval, QRS complex, ST segment, T wave, QT interval, and corrected QT interval (QTc).

#### 4.4.6. Ophthalmologic Examination

The ophthalmologic examination was conducted 28 days after the first administration. Inspection items included, but were not limited to, visual response, eyelid, conjunctiva, cornea, sclera, iris, pupil, lens, vitreous, and fundus.

#### 4.4.7. Hematology and Serum Biochemistry Indices

Approximately 2 mL of dog blood through a peripheral vein puncture from the limbs was taken on day 28 (administration period endpoint). To obtain the blood cell counts, the blood sample was transferred into a CBC bottle with EDTA-2 K (3 mL, Vacutainer, BD, USA). Parameters like red blood cell counts (RBC), white blood cell counts (WBC), and hemoglobin (HGB), etc. [23] were collected through an XT2000i automatic blood cell analyzer (SYSMEX, Hyogo, Japan). To obtain the coagulation function index, the withdrawn blood samples were anticoagulated with sodium citrate (1:9) to separate plasma and analyzed with a Coatron 1800 automatic blood coagulation analyzer (TECO, Hilden, Germany). Parameters like prothrombin time (PT) and activated partial thromboplastin time (APTT) were calculated.

For the measurement of serum biochemistry, at the same time point, another 3 mL of blood samples were withdrawn. Samples were analyzed through an AU480 Automatic Biochemical Analyzer (Beckman, Brea, CA, USA) and a Medica EasyLyte PLUS Electrolyte Analyzer (Medica, Bedford, MA, USA) to calculate parameters like creatinine (CREA), blood urea nitrogen (BUN), blood glucose (GLU), etc.

#### 4.4.8. Bone Marrow Smear

During the animal dissection, bone marrow was collected from the sternum and spread onto a slide. The bone marrow was then subjected to cytology examination after staining with Wright’s stain.

#### 4.4.9. Gross Necropsy

On day 28, all surviving animals were subjected to a gross necropsy. Anesthesia was induced in the beagle dogs using 3% pentobarbital sodium. Once the corneal reflex disappeared, the animals were euthanized by bleeding through the femoral artery, followed by a necropsy. The gross necropsy included an examination of the body surface, cavity, musculoskeletal system, cranial cavity, thoracic cavity, abdominal cavity, pelvic cavity, and other areas [24]. If any abnormalities were found, the abnormal tissue or organ was recorded promptly.

#### 4.4.10. Organ/Body Weight Coefficient

During dissection, the animal body weight and the organ weight of the brain, thyroid, heart, liver, spleen, kidneys, adrenal glands, thymus, testis, and epididymis were recorded for the calculation of the organ/body weight coefficient (O/B coefficient).

#### 4.4.11. Histological Study

Minor brains were fixed in 10% neutral phosphate-buffered formalin and embedded in paraffin. Sections were made and stained with hematoxylin and eosin (H&E) for microscopic examination.

### 4.5. Toxicokinetics Study

#### 4.5.1. Blood Samples Collection

1 mL of blood was withdrawn from veins of limbs at 0.5, 1, 2, 4, 8, and 24 h on day 1 and day 28, and added to a vacuum blood collection tube anticoagulated by heparin sodium, centrifuged at 3500 rpm for 10 min at 4 °C, and the plasma was separated into EP tubes and stored in a −80 °C refrigerator.

#### 4.5.2. Sample Pretreatment

50 μL of plasma was added with 50 μL of 80% acetonitrile solution (*w*/*v*) and 150 μL of the internal standard solution (2 ng/mL), with 5 times the volume of ethyl acetate further added, vortexed for 3 min, and centrifuged for another 10 min at 2000× *g*. The supernatant was removed and evaporated with nitrogen. The resultant was redissolved with the mobile phase for further analysis.

#### 4.5.3. Analytical Method

An ultra-high performance liquid chromatography (Agilent Technologies, Inc., Santa Clara, CA, USA)—quadrupole linear ion trap mass spectrometry (Danaher Corporation, Washington, DC, USA) (UPLC-QqLIT-MS) equipped with Agilent HC-C8 column (4.6 mm × 150 mm, 5 μm) (Agilent Technologies, Inc., Santa Clara, CA, USA) was used for this study. The mobile phase was 10 mM Ammonium Acetate-Acetonitrile (30:70), the flow rate was 0.5 mL/min, and the column temperature was set as 40 °C. The ionization mode was set as ESI positive ion mode, and multiple reaction monitoring (MRM) scanning mode was conducted, while 205.1→178.2 was monitored for the LVM ion pair, 296.1→264.1 for the mebendazole ion pair.

#### 4.5.4. Calibration Curve and Quality Control Samples

To analyze the plasma samples, the calibration curve was freshly prepared in each batch run. Seven series of calibration curves were generated by adding 50 µL of each working standard solution to 50 µL of blank plasma to reach the concentrations of 0.5, 1.0, 5.0, 2.0, 10.0, 50.0, 100.0, and 200.0 ng/mL, along with 150 µL of the internal standard (IS). The concentrations of the samples were calculated using the linear equation obtained from regression analysis of the calibration curve. The QC samples of plasma were well prepared to reach concentrations at 0.5 ng/mL for the lower limit of quantification (LLOQ), 1.5 ng/mL for low quality control (LQC), 10.0 ng/mL for medium quality control (MQC), and 150.0 ng/mL for high quality control (HQC).

#### 4.5.5. Method Validation

The method validations included assessing selectivity, linearity, accuracy and precision, recovery, dilution integrity, carry-over, freeze and thaw stability, and post-preparative stability. Selectivity was assessed by analyzing six different sources of blank plasma to evaluate any interfering components at the retention time for LVM and IS [25,26,27]. For linearity, a calibration curve was constructed using seven standard points ranging from 0.5–200.0 ng/mL for LVM in plasma. The coefficient of determination (R2) should be greater than 0.99. The reproducibility of the calibration curve was evaluated on different days (n = 3). Accuracy and precision were determined by analyzing LLOQ, LQC, MQC, and HQC samples with five replicates at each concentration. The inter batch for accuracy and precision was conducted on 3 different days. Recovery was determined by comparing the response of LVM for LQC and HQC samples from five replicates of pre-extracted samples to the response obtained from post-extraction. Dilution integrity was assessed to demonstrate that a sample diluted to a concentration within the working range can still provide a reliable result. The samples were analyzed and calculated using the dilution factor. Carry-over was examined by injecting extracted blank samples after the extracted ULOQ. Freeze and thaw stability were evaluated by preparing LQC and HQC samples, storing them at −80 °C, and thawing them at room temperature. The freeze–thaw cycle was repeated three times for plasma samples, and the concentrations were compared with the prepared samples after each cycle. Post-preparative stability was determined by preparing three replicates of LQC and HQC samples, evaluating them with freshly prepared samples, and then keeping them for 24 h before re-evaluating and comparing them with the freshly prepared samples [28].

### 4.6. Statistical Analysis

All data generated in the study, except those directly collected by computers or automated instruments, are recorded on forms or recording paper. Records and changes were made according to relevant SOPs, and raw data should be collated and bound at all times. All recorded data are verified by another person. Secondary data to be entered are backed up by Excel software (Microsoft Excel^®^ 2013, Redmond, WA, USA). Secondary data to be entered are backed up by Excel software, and the entered data are also verified by another person and confirmed by the SD to ensure the reliability of the data.

SPSS 21.0 (SPSS Inc., Chicago, IL, USA) was used in this study for the statistical analysis. Body weight, body temperature, electrocardiogram, hematological index, blood biochemical index, organ weight, and organ coefficient were counted according to the following methods, and the results were expressed as mean ± standard deviation. For normally distributed data, Levene’s test was used for homogeneity of variance. When the variance is homogeneous (*p* > 0.05) and there is a statistical difference in one-way analysis of variance (*p* ≤ 0.05), Dunnett’s test was used for comparison between groups; when the variance is unequal (*p* ≤ 0.05) When *p* ≤ 0.05), the comparison between groups was performed using Tamhane’s T2 test. Non-normally distributed data were analyzed using the nonparametric Kruskal-Wallis H test.

## 5. Conclusions

Overall, combined with the full-text data, a NOAEL of LVM was set as 30 mg/kg/day for the male beagle dog (12-fold equivalent to the human clinical dose). At this exposure level, LVM did not show any significant toxicological effect on beagle dogs, and repeated exposure to LVM at the toxic dose for 4 weeks shows no bioaccumulation. The results of this repeated toxicity test and toxicokinetics study can provide guidance for the clinical application of LVM with significantly good safety profiles.

## Figures and Tables

**Table 1:** Parameters of hematological examination (mean ± SD, n = 5).

Parameters	0 mg/kg	5 mg/kg	15 mg/kg	30 mg/kg
RBC (×10^12^/L)	6.64 ± 0.72	6.40 ± 0.63	6.53 ± 0.47	6.10 ± 0.14
WBC (10^9^/L)	15.09 ± 2.34	12.00 ± 2.10 *	13.04 ± 1.12	10.31 ± 0.99 **
HGB (g/L)	144.40 ± 13.26	144.40 ± 19.72	141.80 ± 10.94	130.60 ± 5.27
HCT (%)	42.08 ± 3.42	42.58 ± 5.94	40.72 ± 2.94	37.30 ± 1.85
MCV (fL)	63.52 ± 2.28	66.30 ± 3.35	62.40 ± 1.43	61.10 ± 1.76
MCH (pg)	21.76 ± 0.61	22.50 ± 1.10	21.72 ± 0.48	21.40 ± 0.50
NEUT (%)	55.34 ± 4.41	55.06 ± 4.81	56.08 ± 4.83	55.66 ± 6.49
LYMPH (%)	35.84 ± 4.95	34.36 ± 5.74	32.74 ± 5.53	32.86 ± 8.35
MONO (%)	5.44 ± 1.60	7.42 ± 1.17	7.78 ± 2.31	9.18 ± 1.97 *
EO (%)	3.24 ± 0.72	2.94 ± 1.18	3.16 ± 0.85	2.04 ± 1.07
BASO (%)	0.14 ± 0.05	0.22 ± 0.08	0.24 ± 0.15	0.26 ± 0.15
NEUT (10^9^/L)	8.36 ± 1.48	6.55 ± 0.839 *	7.30 ± 0.85	5.74 ± 0.92 **
LYMPH (×10^9^/L)	5.40 ± 0.98	4.19 ± 1.27	4.27 ± 0.77	3.39 ± 0.92 *
MONO (×10^9^/L)	0.82 ± 0.24	0.88 ± 0.13	1.02 ± 0.34	0.94 ± 0.18
EO (×10^9^/L)	0.50 ± 0.19	0.36 ± 0.16	0.42 ± 0.13	0.21 ± 0.11 *
BASO (×10^9^/L)	0.02 ± 0.01	0.03 ± 0.01	0.03 ± 0.02	0.03 ± 0.02
PLT (×10^9^/L)	307.68 ± 26.30	306.16 ± 81.07	273.12 ± 119.42	331.56 ± 72.21
PT (sec)	8.46 ± 2.80	7.08 ± 0.38	7.18 ± 0.20	7.24 ± 0.76
APTT (sec)	24.62 ± 1.35	24.62 ± 1.39	23.08 ± 1.56	24.04 ± 1.34

*: *p* < 0.05, **: *p* < 0.01 compared with the 0 mg/kg group over the same period.

**Table 2:** Major parameters of blood biochemical examination (mean ± SD, n = 5).

Parameters	0 mg/kg	5 mg/kg	15 mg/kg	30 mg/kg
CREA (μM)	54.22 ± 7.23	58.26 ± 10.31	57.38 ± 5.90	57.14 ± 3.73
BUN (mM)	3.34 ± 0.60	4.04 ± 1.51	4.14 ± 0.88	4.32 ± 0.81
GLU (mM)	7.08 ± 0.28	6.52 ± 0.62	6.54 ± 0.34	6.68 ± 0.49
TP (g/L)	60.12 ± 6.04	60.10 ± 4.96	60.20 ± 3.80	60.14 ± 2.62
ALB (g/L)	28.32 ± 1.61	28.88 ± 1.54	29.36 ± 2.82	28.96 ± 2.49
TBIL (μM)	2.78 ± 0.22	2.98 ± 0.19	2.78 ± 0.37	2.40 ± 1.56
AST (U/L)	32.22 ± 3.60	29.02 ± 3.94	43.04 ± 22.65	50.58 ± 50.16
ALT (U/L)	35.68 ± 7.47	27.64 ± 5.01	31.02 ± 2.87	43.14 ± 19.47
CK (U/L)	238.38 ± 79.69	197.64 ± 69.00	258.64 ± 84.01	598.26 ± 828.12
ALP (U/L)	156.72 ± 99.59	234.92 ± 76.42	138.86 ± 54.65	160.86 ± 29.12
GGT (U/L)	2.44 ± 0.52	2.16 ± 0.72	2.64 ± 0.95	2.36 ± 0.44
TG (mM)	0.58 ± 0.07	0.76 ± 0.06 **	0.72 ± 0.11 *	0.79 ± 0.08 **
CHOL (mM)	4.06 ± 0.55	6.27 ± 1.14	6.59 ± 1.24	8.92 ± 2.54 ***
Na^+^ (mM)	139.62 ± 0.50	141.80 ± 1.08 **	139.14 ± 1.13	139.70 ± 1.08
K^+^ (mM)	4.69 ± 0.15	4.97 ± 0.31	4.47 ± 0.36	4.71 ± 0.43
Cl^-^ (mM)	114.24 ± 1.25	115.12 ± 0.51	115.68 ± 0.54	113.88 ± 1.01

*: *p* < 0.05, **: *p* < 0.01, ***: *p* < 0.001 compared with the 0 mg/kg group over the same period.

**Table 3:** Major parameters of bone marrow examination (mean ± SD, n = 3).

Parameters	0 mg/kg	5 mg/kg	15 mg/kg	30 mg/kg
Myeloblast (%)	1.33 ± 0.94	1.67 ± 0.47	1.33 ± 1.25	0.33 ± 0.47
Progranulocyte (%)	7.00 ± 2.65	5.00 ± 4.36	5.33 ± 2.51	10.3 ± 3.51
Progranulocyte (%)	3.83 ± 1.61	3.33 ± 1.53	2.83 ± 0.76	12.8 ± 3.88
Metamylocyte (%)	17.5 ± 5.50	18.3 ± 2.57	13.3 ± 1.04	13.3 ± 1.15
Eosinophils (%)	0.00 ± 0.00	0.00 ± 0.00	0.17 ± 0.29	0.00 ± 0.00
Eosinophils (%)	0.00 ± 0.00	0.00 ± 0.00	0.00 ± 0.00	0.00 ± 0.00
Pronormoblasts (%)	0.67 ± 0.76	0.50 ± 0.00	0.67 ± 0.76	1.80 ± 0.76
Prorubricyte (%)	2.67 ± 1.61	4.50 ± 2.00	2.17 ± 1.76	2.67 ± 1.04
Polychromatic (%)	8.83 ± 3.51	12.7 ± 4.19	9.67 ± 1.26	12.2 ± 2.25
Metarubricyte (%)	36.3 ± 7.64	28.2 ± 3.79	36.5 ± 3.50	33.7 ± 11.7
Myeloid erythroid ratio	0.46 ± 0.14	0.53 ± 0.04	0.45 ± 0.03	0.50 ± 0.25
Metarubricyte (%)	5.17 ± 2.75	4.67 ± 1.61	4.17 ± 3.25	1.83 ± 0.58
Monocyte (%)	3.67 ± 2.02	1.00 ± 0.87	3.00 ± 2.18	1.33 ± 0.58
Plasmocyte (%)	0.17 ± 0.29	0.50 ± 0.00	0.33 ± 0.29	0.33 ± 0.29

**Table 4:** Organ weight/body weight (O/B) coefficient (mean ± SD, n = 5).

Organ	0 mg/kg	5 mg/kg	15 mg/kg	30 mg/kg
Brain	0.961 ± 0.102	0.906 ± 0.045	0.893 ± 0.043	0.897 ± 0.075
Thyroid	0.013 ± 0.002	0.012 ± 0.004	0.009 ± 0.001	0.010 ± 0.002
Heart	0.689 ± 0.086	0.718 ± 0.019	0.754 ± 0.009	0.765 ± 0.155
Liver	3.013 ± 0.077	2.893 ± 0.275	3.082 ± 0.223	3.198 ± 0.274
Spleen	0.325 ± 0.038	0.293 ± 0.059	0.317 ± 0.081	0.281 ± 0.039
Kidneys	0.514 ± 0.024	0.518 ± 0.050	0.494 ± 0.042	0.481 ± 0.050
Adrenal glands	0.015 ± 0.004	0.012 ± 0.002	0.018 ± 0.007	0.019 ± 0.011
Thymus	0.305 ± 0.036	0.279 ± 0.024	0.184 ± 0.065 *	0.155 ± 0.062 *
Testis	0.043 ± 0.031	0.054 ± 0.049	0.101 ± 0.031	0.081 ± 0.033
Epididymis	0.010 ± 0.002	0.013 ± 0.007	0.024 ± 0.006 *	0.022 ± 0.007 *

*: *p* < 0.05 compared with the 0 mg/kg group over the same period.

**Table 5:** Accuracy and precision of LVM in plasma at low quantification limit, low, medium, and high concentrations for intra-batch and inter-batch.

	Concentration of LVM in Plasma (ng/mL)			
	LLOQ (0.5 ng/mL)	LQC (1.5 ng/mL)	MQC (10 ng/mL)	HQC (150 ng/mL)
Mean value of intra-day	0.48	1.61	10.15	145.83
SD	0.01	0.08	0.52	5.23
RSD (%)	2.61	5.04	5.14	3.59
Accuracy (%)	95.4	107.4	101.5	97.2
Mean value of inter-day	0.49	1.58	10.45	146.72
SD	0.06	0.08	0.41	4.18
RSD (%)	12.00	5.16	3.91	2.85
Accuracy (%)	98.3	105.3	104.5	97.8

**Table 6:** Major parameters of toxicokinetics of LVM in beagle dogs (mean ± SD, n = 5).

Dosage	Day	T_max_ (h)	C_max_ (ng/mL)	AUC_0-t_ (h*ng/mL)
5 mg/kg	1	0.90 ± 0.65	994.68 ± 435.86	2999.50 ± 778.80
	28	0.50 ± 0.00	1304.28 ± 367.21	4851.26 ± 1299.42
15 mg/kg	1	1.00 ± 0.55	3568.07 ± 1337.19	12,326.52 ± 3144.35
	28	0.50 ± 0.00	5027.63 ± 739.03	15,990.54 ± 6690.98
30 mg/kg	1	1.60 ± 1.47	8102.45 ± 4913.85	30,387.45 ± 9603.64
	28	1.90 ± 1.34	6275.85 ± 5456.97	32,740.81 ± 23,859.51

## Data Availability

Data is contained within the article and Supplementary Materials.

## Supplementary Materials

The following supporting information can be downloaded at: https://www.mdpi.com/article/10.3390/ph17010141/s1, Table S1: The individual clinical observation; Table S2: The ECG values of individual experimental animals; Table S3: Major parameters of bone marrow examination.
