# Supplementary material for: Toxicity and Toxicokinetics of a Four-Week Repeated Gavage of Levamisole in Male Beagle Dogs: A Good Laboratory Practice Study
Source: Pharmaceuticals (Basel). 2024 Jan 22;17(1):141. doi: 10.3390/ph17010141 (PMC10819294; doi:10.3390/ph17010141)
Supplement: Supplementary file 1 [file pharmaceuticals-17-00141-s001.zip › pharmaceuticals-2801490-supplementary.pdf]

Table S1 The individual clinical observation

**Dosage (mg/kg)**	**No.**	**D1**	**D2**	**D3**	**D4**	**D5**	**D6**	**D7**	**D8**	**D9**	**D10**	**D11**	**D12**	**D13**	**D14**
0	1	√	√	√	√	√	√	√	√	√	√	√	√	√	√
	2	√	√	√	√	√	√	√	√	√	√	√	√	√	√
	3	√	√	√	√	√	√	√	√	√	√	√	√	√	√
	4	√	√	√	√	√	√	√	√	√	√	√	√	√	√
	5	√	√	√	√	√	√	√	√	√	√	√	√	√	√
5	6	√	√	√	√	√	√	√	√	√	√	√	√	√	√
	7	√	√	√	√	√	√	√	√	√	√	√	√	√	√
	8	√	√	√	√	√	√	√	√	√	√	√	√	√	√
	9	√	√	√	√	√	√	√	√	√	√	√	√	√	√
	10	√	√	√	√	√	√	√	√	√	√	√	√	√	√
15	11	√	√	√	√	√	1	√	√	1	√	√	1	√	√
	12	√	√	√	√	√	1	1	√	1	√	√	√	√	√
	13	√	√	√	√	√	√	√	√	√	√	√	√	√	√
	14	√	√	1	√	√	√	√	√	√	√	√	√	√	√
	15	√	√	√	√	√	√	√	√	√	√	√	1	√	√
30	16	1	1	1	1	1	1	1	1	1	1	1	1	√	1
	17	1	1	1	1,3	√	1	1	1	1	1	1	1	1	2
	18	1,2,3	√	1,4	1,2,4	1	1	1	1,2	1,2	2	2	2	1,2	1,2
	19	1	√	1	√	1,2	√	1	1	1	2	√	√	√	√
	20	1	√	√	√	7	1	1	1	√	√	1	√	√	√

√ : Normal; 1: Vomiting; 2: Salivation; 3: Tremor; 4: Loose stool; 5: Hypoactivity; 6 Vomiting, head tilt, gait disturbance; 7: Vomiting, shortness of breath; 8: Tremor, vomiting, head droop; 9 convulsions, vomiting; 10: death.

Table S1 The individual clinical observation (Con't)

Dosage (mg/kg)	No.	D15	D16	D17	D18	D19	D20	D21	D22	D23	D24	D25	D26	D27	D28
0	1	√	√	√	√	√	√	√	√	√	√	√	√	√	√
	2	√	√	√	√	√	√	√	√	√	√	√	√	√	√
	3	√	√	√	√	√	√	√	√	√	√	√	√	√	√
	4	√	√	√	√	√	√	√	√	√	√	√	√	√	√
	5	√	√	√	√	√	√	√	√	√	√	√	√	√	√
5	6	√	√	√	√	√	√	√	√	√	√	√	√	√	√
	7	√	√	√	√	√	√	√	√	√	√	√	√	√	√
	8	√	√	√	√	√	√	√	√	√	√	√	√	√	√
	9	√	√	√	√	√	√	√	√	√	√	√	√	√	√
	10	√	√	√	√	√	√	√	√	√	√	√	√	√	√
15	11	√	√	√	√	√	√	√	√	√	√	√	√	√	1
	12	√	√	√	1	√	√	√	√	√	√	√	√	1	1
	13	√	√	√	√	√	√	√	√	√	√	√	√	√	√
	14	√	√	√	√	√	√	√	√	√	√	√	√	√	√
	15	1	√	√	1	√	√	√	√	√	1,2	2	√	√	√
30	16	1	√	6	1,2	2	1	√	√	√	√	√	2		1
	17	1	2	2	2	2	√	2	2	2	2,3	2	2	2	2
	18	1,2	1,2	2	2,4	2	1,2	2	2	√	2	√	2	2	9
	19	1,2	2	2	7	2	2	2,3	2	2	2	8	√	2	2,5
	20	√	√	√	1	√	√	√	√	√	√	√	√	√	√

√ : Normal; 1: Vomiting; 2: Salivation; 3: Tremor; 4: Loose stool; 5: Hypoactivity; 6 Vomiting, head tilt, gait disturbance; 7: Vomiting, shortness of breath; 8: Tremor, vomiting, head droop; 9 convulsions, vomiting; 10: death.

Table S2 The ECG values of individual experimental animals

Dosage (mg/kg)	No.	HR (bpm)	PR (ms)	QRS (ms)	QT (ms)	QTc (ms)	ST (mV)	T (mV)
0	1	134	98	41	192	292	5	25
	2	132	94	42	176	258	-3	-2
	3	136	92	40	224	340	6	4
	4	97	95	41	202	259	15	49
	5	145	105	44	174	271	2	-30
5	6	165	93	49	199	324	0	24
	7	150	89	38	189	296	1	20
	8	133	100	46	192	283	-2	26
	9	100	102	45	205	268	7	-5
	10	108	86	41	188	254	-2	-9
15	11	125	94	44	184	270	-7	-20
	12	109	106	38	135	227	-1	1
	13	166	87	43	144	241	-1	4
	14	117	102	37	197	274	3	23
	15	160	106	38	184	299	15	-7
30	16	144	79	41	185	284	1	-17
	17	138	89	48	207	310	5	11
	18	99	96	50	217	282	-8	-28
	19	130	80	42	184	271	-1	19
	20	105	112	40	188	243	-1	-21

Table S3 Major parameters of bone marrow examination

parameters	control			5mg/kg			15mg/kg		
Myeloblast (%)	2(1.0)	2(1.0)	0(0)	2(1.0)	2(1.0)	1(0.5)	1(0.5)	0(0)	3(1.5)
Progranulocyte (%)	6（3.0）	10(5.0)	5(2.5)	3(1.5)	2(1.0)	10(5.0)	8(4.0)	3(1.5)	5(2.5)
Progranulocyte (%)	9（4.5）	4(2.0)	10(5.0)	6（3）	4(2.0)	10(5.0)	7(3.5)	6（3）	4(2.0)
Metamylocyte（%）	24（12.0）	46(23.0)	35（17.5）	31(15.5)	41(20.5)	38(19.0)	25(12.5)	29(14.5)	26(13.0)
Eosinophils (%)	0(0)	0(0)	0(0)	0(0)	0(0)	0(0)	0(0)	1(0.5)	0(0)
Eosinophils (%)	0(0)	0(0)	0(0)	0(0)	0(0)	0(0)	0(0)	0(0)	0(0)
Pronormoblasts (%)	3(1.5)	1(0.5)	0(0)	1(0.5)	1(0.5)	1(0.5)	1(0.5)	0(0)	3(1.5)
Prorubricyte (%)	9(4.5)	3(1.5)	4(2.0)	13(6.5)	9(4.5)	5(2.5)	8(4.0)	1(0.5)	4(2.0)
polychromatic （%）	25(12.5)	11（5.5）	17(8.5)	21(10.5)	20(10.0)	35(17.5)	22(11.0)	19(9.5)	17(8.5)
Metarubricyte (%)	76(38.0)	86(43.0)	56(28.0)	51(25.5)	65(32.5)	53(26.5)	65(32.5)	78(39.0)	76(38.0)
Myeloid erythroid ratio	0.68	0.83	1.22	1.15	1	1	0.88	0.84	0.95
Metarubricyte (%)	5(2.5)	10(5.0)	16(8.0)	13(6.5)	8(4.0)	7(3.5)	15(7.5)	8(4.0)	2(1.0)
Monocyte (%)	5(2.5)	5(2.5)	12(6.0)	1(0.5)	1(0.5)	4(2.0)	4(2.0)	11(5.5)	3(1.5)
plasmocyte（%)	0(0)	0(0)	1(0.5)	1(0.5)	1(0.5)	1(0.5)	1(0.5)	1(0.5)	0(0)
Monocyte (%)	30(15.0)	5(2.5)	2(1.0)	25(12.5)	12(6.0)	13(6.5)	2(1.0)	10(5.0)	13(7.5)
